# Structures of *Brucella ovis* leucine-, isoleucine-, valine-, threonine- and alanine-binding protein reveal a conformationally flexible peptide-binding cavity

**DOI:** 10.1107/S2053230X24007027

**Published:** 2024-08-23

**Authors:** Graham Chakafana, Reghan Boswell, Andrew Chandler, Krishelle A. Jackson, Sanai Neblett, Tyler Postal, Sandhya Subramanian, Jan Abendroth, Peter J. Myler, Oluwatoyin A. Asojo

**Affiliations:** a Department of Chemistry and Biochemistry Hampton University Hampton VA23668 USA; b Center for Global Infectious Disease Research Seattle Children’s Research Institute 307 Westlake Avenue North Suite 500 Seattle WA98109 USA; c Seattle Structural Genomics Center for Infectious Disease (SSGCID), Seattle, Washington, USA; d UCB BioSciences, Bainbridge Island, WA98110, USA; e Departments of Pediatrics, Global Health, and Biomedical Informatics and Medical Education University of Washington Seattle Washington USA; f Dartmouth Cancer Center Dartmouth College One Medical Center Drive Lebanon NH03756 USA; University of York, United Kingdom

**Keywords:** SSGCID, structural genomics, brucellosis, epididymitis, transport protein, drug repurposing

## Abstract

*Brucella ovis* is an etiologic agent of ovine epididymitis and brucellosis that causes global devastation in sheep, rams, goats, small ruminants and deer. There are no cost-effective methods for the worldwide eradication of ovine brucellosis. *B. ovis* and other protein targets from various *Brucella* species are currently in the pipeline for high-throughput structural analysis at the Seattle Structural Genomics Center for Infectious Disease (SSGCID), with the aim of identifying new therapeutic targets. Furthermore, the wealth of structures generated are effective tools for teaching scientific communication, structural science and biochemistry. One of these structures, *B. ovis* leucine-, isoleucine-, valine-, threonine- and alanine-binding protein (*Bo*LBP), is a putative periplasmic amino acid-binding protein. *Bo*LBP shares less than 29% sequence identity with any other structure in the Protein Data Bank. The production, crystallization and high-resolution structures of *Bo*LBP are reported. *Bo*LBP is a prototypical bacterial periplasmic amino acid-binding protein with the characteristic Venus flytrap topology of two globular domains encapsulating a large central cavity containing the peptide-binding region. The central cavity contains small molecules usurped from the crystallization milieu. The reported structures reveal the conformational flexibility of the central cavity in the absence of bound peptides. The structural similarity to other LBPs can be exploited to accelerate drug repurposing.

## Abstract 2

*Brucella ovis* leucine-, isoleucine-, valine-, threonine- and alanine-binding protein structures have a prototypical bacterial periplasmic amino acid-binding protein topology with a conformationally flexible peptide-binding cavity in the absence of peptide.

## 1. Introduction

Brucellosis is highly contagious and affects both economically important livestock and wild animals (Ducrotoy *et al.*, 2017 ▸; Godfroid, Garin-Bastuji *et al.*, 2013 ▸; Godfroid *et al.*, 2011 ▸; Megersa *et al.*, 2011 ▸; Rossetti *et al.*, 2022 ▸). Even when not resulting in significant zoonotic disease, as in the case of *Brucella ovis* (sheep and rams), brucellosis is economically devastating globally (Peck & Bruce, 2017 ▸; Franc *et al.*, 2018 ▸). While brucellosis has been eradicated in cattle and small ruminants in some industrialized countries, it remains endemic globally within many animal hosts (Moreno, 2014 ▸). Current control approaches for brucellosis include vaccination, education and basic hygiene. However, these strategies have yet to reduce the disease burden successfully due to the high costs, the ineffectiveness of current antibiotics in the latter stages of brucellosis and other factors (Ariza *et al.*, 2007 ▸; Franc *et al.*, 2018 ▸). Notably, current vaccines are species-specific and are devastating to pregnant livestock, and some animal care practices of rural dwellers and nomadic groups are incompatible with controlling brucellosis in humans and livestock (Ducrotoy *et al.*, 2017 ▸; Godfroid, Al Dahouk *et al.*, 2013 ▸).

*B. ovis* is nonpathogenic in humans but is devastating globally to sheep, rams, goats, small ruminants and deer by causing ovine epididymitis (Rossetti *et al.*, 2022 ▸). Similarly, *B. melitensis*, which causes fatal zoonotic disease in humans, also causes ovine epididymitis (Rossetti *et al.*, 2022 ▸). *Brucella* are classified as category B infectious agents that can be aerosolized, and these small Gram-negative, facultative coccobacilli were the first bacterial agent to successfully be developed for biological warfare by the United States (de Figueiredo *et al.*, 2015 ▸; Riedel, 2004 ▸). There is a continued need for brucellosis treatments in infected people and livestock. New approaches include the rational design or repurposing of small molecules that target proteins that are vital for bacterial survival. Towards these ends, the Seattle Structural Genomics Center for Infectious Disease (SSGCID) has determined the crystal structures of over 120 potential target proteins from different *Brucella* species. These structures provide a wealth of data for functionally and structurally characterizing *Brucella* proteins that are potential therapeutic targets and provide insights into fundamental mechanisms that can be used for drug discovery. These structures are used to engage undergraduates in structure analysis and scientific communication (Brooks *et al.*, 2022 ▸; Davidson *et al.*, 2022 ▸; Maddy *et al.*, 2022 ▸; Porter *et al.*, 2022 ▸; Beard, Bristol *et al.*, 2022 ▸; Beard, Subramanian *et al.*, 2022 ▸). Here, we present high-resolution crystal structures of *B. ovis* leucine-, iso­leucine-, valine-, threonine- and alanine-binding protein (*Bo*LBP). *Bo*LBP is a putative periplasmic amino acid-binding protein with less than 29% sequence identity to any previously reported structure. We report high-resolution structures of *Bo*LBP in orthorhombic and monoclinic space groups that reveal a prototypical periplasmic amino acid-binding protein.

## 2. Materials and methods

### 2.1. Macromolecule production

Cloning, expression and purification followed standard protocols as described previously (Bryan *et al.*, 2011 ▸; Choi *et al.*, 2011 ▸; Serbzhinskiy *et al.*, 2015 ▸; Brooks *et al.*, 2022 ▸; Davidson *et al.*, 2022 ▸; Maddy *et al.*, 2022 ▸; Porter *et al.*, 2022 ▸). The leucine-, isoleucine-, valine-, threonine- and alanine-binding protein from *B. ovis* (*Bo*LBP; UniProt A0A0H3ATZ3) encoding amino acids 83–471 was PCR-amplified from cDNA using the primers given in Table 1 ▸ and cloned by ligation-independent cloning (LIC), encoding a non­cleavable hexahistidine tag (MAHHHHHH-ORF; Aslanidis & de Jong, 1990 ▸; Choi *et al.*, 2011 ▸). The plasmid DNA was transformed into chemically competent *Escherichia coli* BL21(DE3)R3 Rosetta cells. The plasmid containing *Bo*LBP underwent expression testing, and 2 l of culture was grown using auto-induction medium (Studier, 2005 ▸) in a LEX Bio­reactor (Epiphyte Three Inc.), which allows the controlled expression of proteins, as described previously (Serbzhinskiy *et al.*, 2015 ▸). The expression clone BrovA.17370.a.B2.GE38164 is available at https://www.ssgcid.org/available-materials/expression-clones/.

*Bo*LBP was purified using the established two-step SSGCID pipeline protocol consisting of an immobilized metal (Ni^2+^) affinity chromatography (IMAC) step and size-exclusion chromatography (SEC). All chromatography runs were performed on an ÄKTApurifier 10 (GE Healthcare) using automated IMAC and SEC programs (Bryan *et al.*, 2011 ▸). Thawed bacterial pellets (∼25 g) were lysed by sonication in 200 ml buffer consisting of 25 m*M* HEPES pH 7.0, 500 m*M* NaCl, 5%(*v*/*v*) glycerol, 0.5%(*w*/*v*) CHAPS, 30 m*M* imidazole, 10 m*M* MgCl_2_, 1 m*M* TCEP, 250 mg ml^−1^ AEBSF, 0.025% sodium azide. After sonication, the crude lysate was clarified with 20 ml Benzonase (25 units ml^−1^) and incubated while mixing at room temperature for 45 min. The lysate was clarified by centrifugation at 10 000 rev min^−1^ for 1 h using a Sorvall centrifuge (Thermo Scientific). The clarified supernatant was then passed over a 5 ml Ni–NTA His-Trap FF column (GE Healthcare) which had been pre-equilibrated with loading buffer consisting of 20 m*M* HEPES pH 7.0, 300 m*M* NaCl, 5%(*v*/*v*) glycerol, 30 m*M* imidazole, 1 m*M* TCEP, 0.025%(*w*/*v*) sodium azide. The column was washed with 20 column volumes (CV) of loading buffer and was eluted with loading buffer plus 250 m*M* imidazole in a linear gradient over 7 CV. The peak fractions were pooled and concentrated to 5 ml. A SEC column (Superdex 75, GE Healthcare) was equilibrated with running buffer [20 m*M* HEPES pH 7.0, 300 m*M* NaCl, 5%(*v*/*v*) glycerol, 1 m*M* TCEP]. The peak fractions were collected and analyzed using SDS–PAGE. *Bo*LBP eluted as a single prominent peak at a molecular mass of ∼49 kDa, and the peak fractions were pooled and concentrated to 49.9 mg ml^−1^ using an Amicon concentrator (Millipore). Aliquots of 200 µl were flash-frozen in liquid nitrogen and stored at −80°C until use. The purified protein BrovA.17370.a.B2.PS02287 is available at https://www.ssgcid.org/available-materials/ssgcid-proteins/.

### 2.2. Crystallization

Purified *Bo*LBP was screened for crystallization in 96-well sitting-drop plates against commercially available screens, including JCSG+ HTS (Rigaku Reagents) and MCSG1 (Microlytic). Vapor-diffusion experiments consisted of equal volumes of protein solution (0.4 µl) and precipitant solution set up at 290 K against an 80 µl reservoir. The crystals were flash-cooled by harvesting and plunging them into liquid nitrogen after passing through Al’s oil or soaking in cryo­solution supplemented with 20%(*v*/*v*) ethylene glycol (Table 2 ▸). Two crystallization conditions were used for data collection. The orthorhombic crystal form was obtained at basic pH using CHES–NaOH and 30%(*w*/*v*) PEG 3000, and was cryoprotected by passing through Al’s oil. Heavy-atom (iodide) phasing was facilitated by the second crystal form, which grew in high salt (1 *M* LiCl) and 30%(*w*/*v*) polyethylene glycol 6000 (PEG 6000). The crystals were subjected to two 30 s soaks in cryo/phasing solution with increasing concentrations of sodium iodide in 20%(*v*/*v*) ethylene glycol. A second structure was obtained from soaking crystals grown in polyethylene glycol 3350 (and 200 m*M* potassium nitrate) overnight with 10 m*M* threonine in the same buffer. The crystal was briefly dipped into cryosolution comprised of the soaking solution and 20%(*v*/*v*) ethylene glycol before vitrification in liquid nitrogen and data collection (Table 2 ▸). Future studies will include co-crystallization and harvesting at different temperatures to identify conditions that may enhance amino-acid binding to *Bo*LBP.

### 2.3. Data collection and processing

For the orthorhombic structure, two data sets were collected: one at 100 K on beamline 21-ID-F at the Advanced Photon Source, Argonne National Laboratory (APS), while the phasing data set was collected on a rotating-anode home source (Table 3 ▸). The monoclinic data set was collected on beamline 21-ID-F at APS. All diffraction data were integrated using *XDS* and reduced using *XSCALE* (Kabsch, 2010 ▸). Raw X-ray diffraction images are available from the Integrated Resource for Reproducibility in Macromolecular Crystallo­graphy at https://www.proteindiffraction.org.

### 2.4. Structure solution and refinement

The structure of the monoclinic conformation was phased *de novo* by single-wavelength anomalous dispersion (SAD) after iodide ion soaks (Abendroth *et al.*, 2011 ▸). Iterative refinement cycles with *Phenix* (Adams *et al.*, 2011 ▸) and manual rebuilding using *Coot* (Emsley & Cowtan, 2004 ▸; Emsley *et al.*, 2010 ▸) generated the model coordinates and structure factors deposited in the Protein Data Bank as entry 4xfk. The orthorhombic structure was phased by molecular replacement using the monoclinic structure as the search model and the *Phaser* software (McCoy *et al.*, 2007 ▸) from the *CCP*4 suite of programs (Collaborative Computational Project, Number 4, 1994 ▸; Krissinel *et al.*, 2004 ▸; Winn *et al.*, 2011 ▸; Agirre *et al.*, 2023 ▸). After iterative refinement cycles with *Phenix* (Adams *et al.*, 2011 ▸) and manual rebuilding using *Coot*, orthorhombic coordinates and structure factors were deposited in the Protein Data Bank as entry 7jfn. Both structures were checked using *MolProbity* (Williams *et al.*, 2018 ▸). The final refinement data are reported in Table 4 ▸.

## 3. Results and discussion

**Figure 1:**
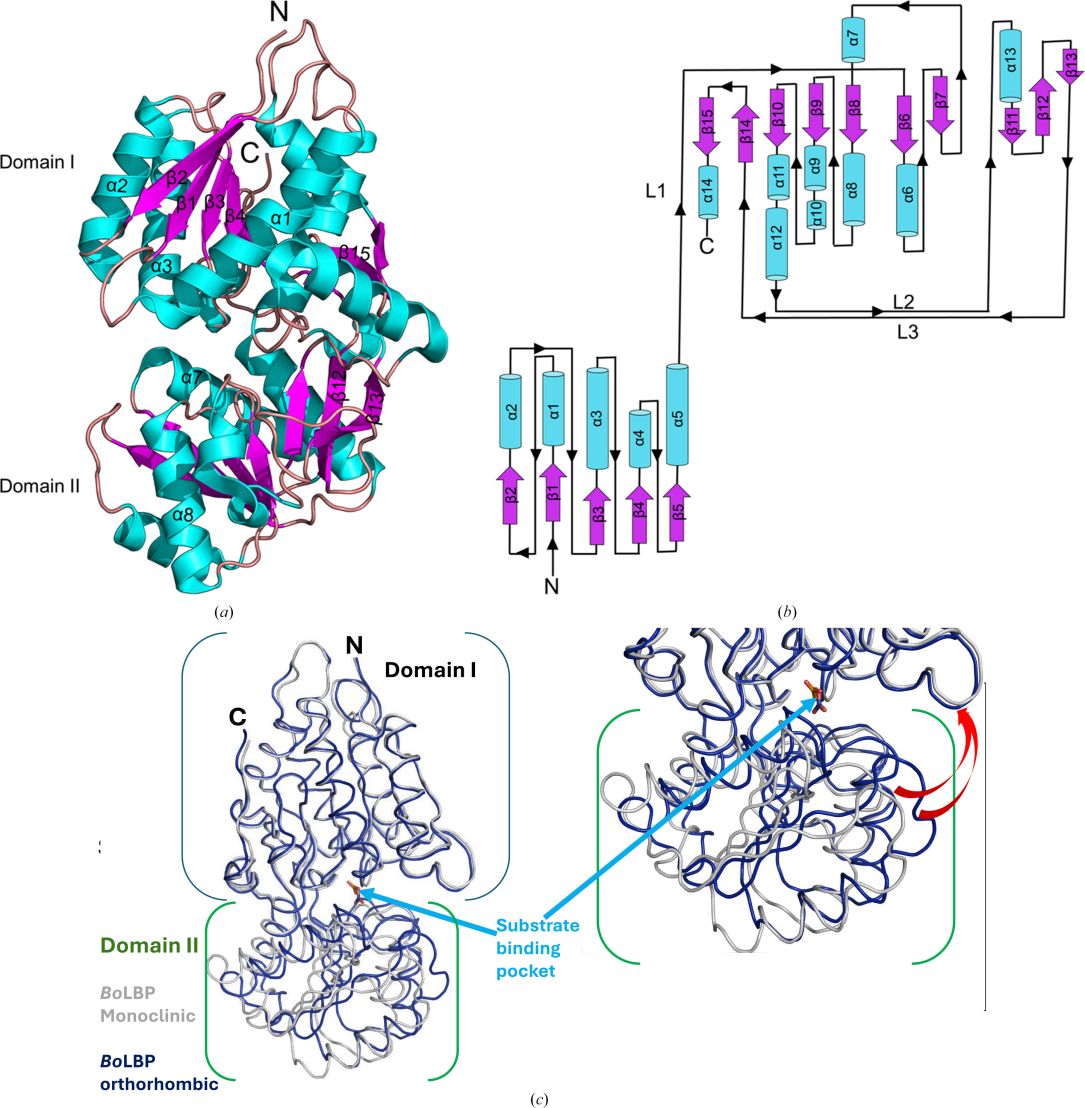
*Bo*LBP structure. (*a*) A ribbon diagram of *Bo*LBP shows 15 β-sheets (purple) and 14 α-helices (cyan). (*b*) The *Bo*LBP topology has two globular domains. α-Helices are labeled α, β-strands are labeled β and the three inter-domain loops are labeled L1, L2 and L3. (*c*) Superposed *Bo*LBP structures. The orthorhombic monomer (blue) has a more open substrate binding cavity than the monoclinic monomer (gray).

*Bo*LBP resembles a prototypical periplasmic amino acid-binding protein, with a bilobate architecture of two major globular domains forming a Venus flytrap conformation around a large central cleft containing the peptide-binding pocket (Trakhanov *et al.*, 2005 ▸). Both domains (domains I and II) have a characteristic α/β fold consisting of a central antiparallel β-sheet flanked by α-helices (Fig. 1 ▸). β-Strands from each sheet run towards the central cleft, exhibiting the characteristic LBP left-handed propeller twist that connects domains I and II through three interdomain loops: loop 1 (L1; residues 224–229), loop 2 (L2; residues 384–393) and loop 3 (L3; residues 423–426) (Fig. 1 ▸*b*). Loops 1 and 3 extend from domain I to domain II, while loop 2 traverses in the opposite direction. Loops 1 and 2 are preceded by a β-sheet strand in one domain and succeeded by an α-helix in the other domain, whereas loop 3 spans strands from both domains (Fig. 1 ▸*b*).

An acetate molecule from the crystallization solution sits in the central cleft in the monoclinic structure determined without soaking with amino acids (PDB entry 4xfk; Supplementary Fig. S1*b*). Threonine does not bind upon soaking the orthorhombic crystals with threonine; instead, a nitrate from the crystallization solution occupies the central binding cavity (PDB entry 7jfn; Supplementary Fig. S1*a*). Both ligands have well ordered electron density in 2*F*_o_ − *F*_c_ maps (Supplementary Figs. S1*b* and S1*c*). The two *Bo*LBP structures are similar, with a root-mean-square deviation (r.m.s.d.) value of 1.49 Å on aligning C^α^ atoms. The main differences in the structures are in domain II, which rotates around the hinge and has a more open central cavity in the orthorhombic structure, with main-chain movements of up to 4.8 Å (Fig. 1 ▸*c*). The differences in the structures are not as large as the conformational changes that are expected when LBP-like proteins transition from an open to a closed conformation upon binding their peptide ligands (Magnusson *et al.*, 2004 ▸). While neither of the *Bo*LBP structures binds an amino acid, both accommodate different ligands from the crystallization solution.

The two structures were compared using *DynDom* (https://dyndom.cmp.uea.ac.uk/dyndom/; Lee *et al.*, 2003 ▸, Qi *et al.*, 2005 ▸). *DynDom* analysis revealed hinge rotation by a 12° angle and conformational plasticity of the ligand-binding cavity in the structures, representing a transition from a ‘closed’ to a ‘semi-closed’ state. Further details of the hinge-bending residues and *DynDom* analysis results are presented in Section S2.

**Figure 2:**
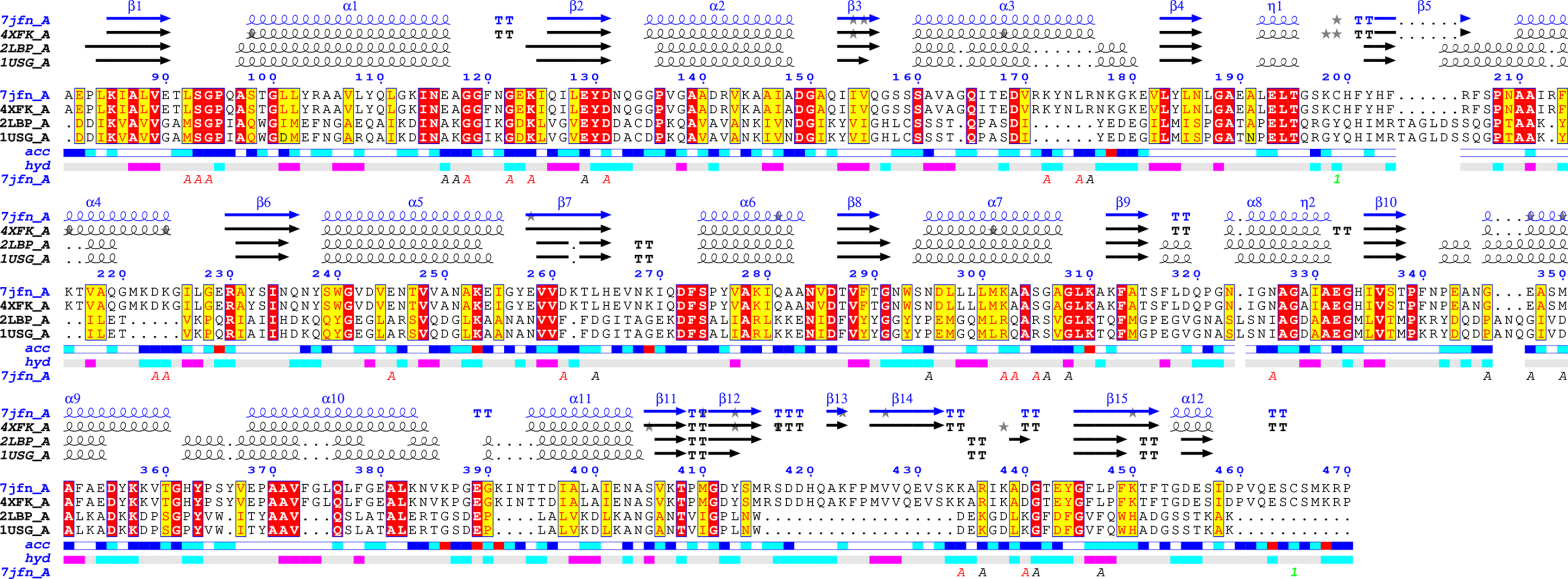
*ENDscript* alignment reveals conserved residues between *Bo*LBP and its closest structural neighbors. Identical and conserved residues are highlighted in red and yellow, respectively. The different secondary-structure elements shown are α-helices (α), 3_10_-helices (η), β-strands (β) and β-turns (TT).

**Figure 3:**
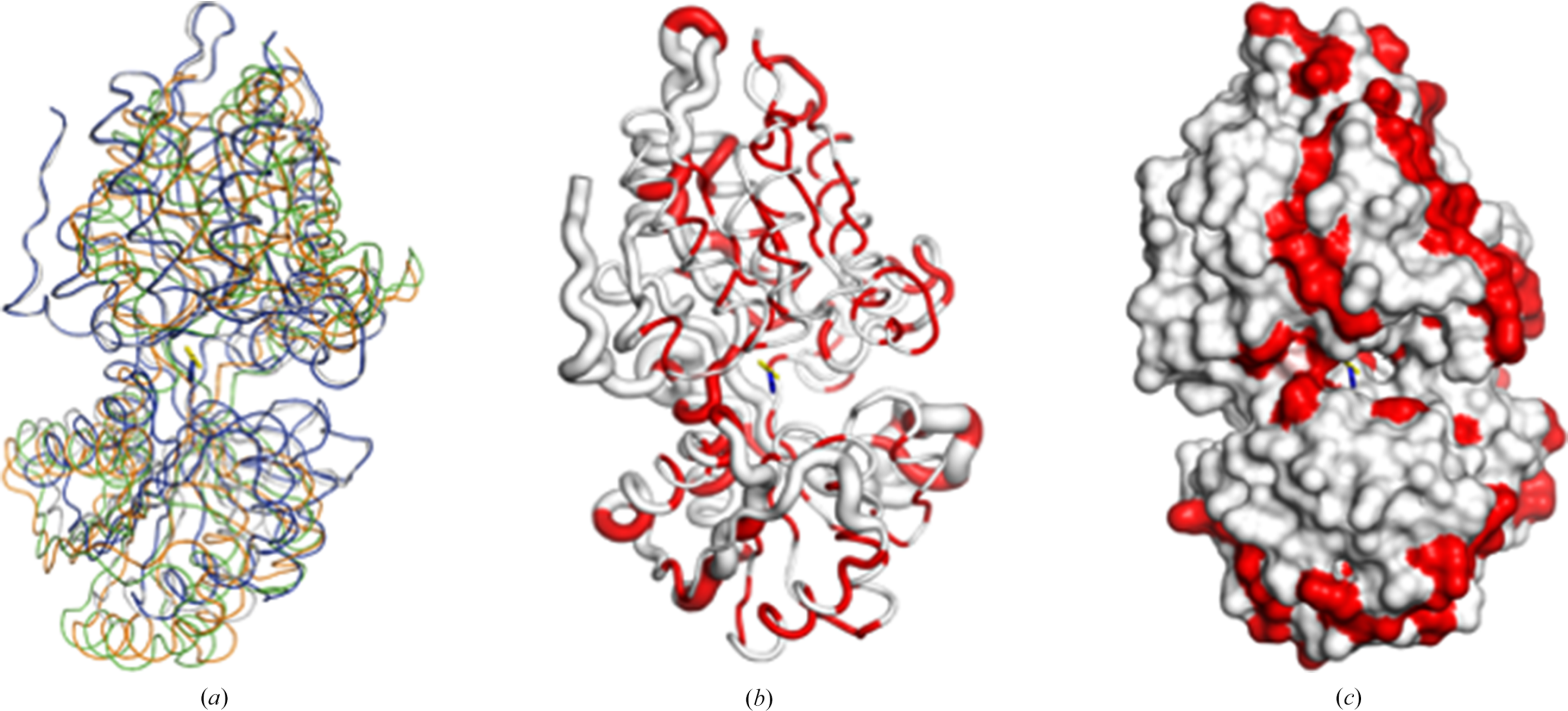
Comparison of *Bo*LBP with its closest structural neighbors. (*a*) The superposed structures show the Venus flytrap motif with a large central cavity. The superposed structures are monoclinic *Bo*LBP (PDB entry 4xfk, gray), orthorhombic *Bo*LBP (PDB entry 7jfn, blue) and *Ec*LBP (PDB entry 1usg, green; PDB entry 2lbp, orange). The acetate (yellow sticks; from PDB entry 4xfk) and nitrate (blue sticks; from PDB entry 7jfn) are shown. (*b*) *ENDscript* coil diagram with thinner ribbons representing more conserved regions and thicker ribbons representing less conserved regions; identical residues in the structures are shown in red. (*c*) *ENDscript* surface diagram: identical residues in the structures are shown in red.

Due to the low sequence similarity of *Bo*LBP to all other reported structures, *ENDscript* (Gouet *et al.*, 2003 ▸; Robert & Gouet, 2014 ▸) analysis was used to identify its closest structural neighbor (Fig. 2 ▸). The analysis identified the closest structural neighbor of *Bo*LBP to be *E. coli* LBP (*Ec*LBP; Sack *et al.*, 1989 ▸), and despite sharing less than 29% sequence similarity both have a similar overall topology (Fig. 2 ▸). Additionally, *Ec*LBP and *Bo*LBP share numerous identical residues (Fig. 2 ▸). Interestingly, *Bo*LBP also has key amino-acid insertions resulting in longer helices and additional strands that were not previously observed in *Ec*LBP (Fig. 2 ▸). The superposed structures show the Venus flytrap conformation around a large central cleft containing the peptide-binding pocket. Structural alignment reveals that the peptide-binding site is occupied by components of the crystallization buffer (acetate and nitrate) in our *Bo*LBP structures (Fig. 3 ▸). The presence of these high-concentration molecules may explain the difficulty of soaking threonine into preformed crystals. *ENDscript* coil analysis shows that the greatest structural difference in the structures lies in the carboxyl-terminus and hinge (Fig. 3 ▸*b*). Identical residues appear interspersed across both domains (Figs. 2 ▸ and 3 ▸). Nonetheless, the similarities between *Bo*LBP, *Ec*LBP and other bacterial LBPs present unique opportunities for rational drug discovery based on the existing data.

## 4. Conclusion

We report two structures of *B. ovis* leucine-, isoleucine-, valine-, threonine- and alanine-binding protein (*Bo*LBP). *Bo*LBP is a prototypical bacterial LBP with additional amino acids inserted outside the central cavity and at the carboxyl-terminus. Both structures exhibit conformational flexibility of *Bo*LBP in the absence of bound amino acids. Despite low sequence similarity, the structures have similarities to bacterial LBPs that can be exploited for future drug-discovery efforts.

## Supplementary Material

PDB reference: *Bo*LBP, 4xfk

PDB reference: 7jfn

Supplementary Figures. DOI: 10.1107/S2053230X24007027/ir5032sup1.pdf

DynDom movie. DOI: 10.1107/S2053230X24007027/ir5032sup2.mov

## Figures and Tables

**Table 1:** Macromolecule-production information

Source organism	*Brucella ovis* (strain ATCC 25840, 63/290, NCTC 10512)
Forward primer	5′-CTCACCACCACCACCACCATATGGCCGAACCGCTGAAGATCG-3′
Reverse primer	5′-ATCCTATCTTACTCACTTAGCCCGGACGCTTCATGGAGC-3′
Cloning vector	BG1861
Expression vector	BG1861
Expression host	BL21(DE3)R3 Rosetta
Complete amino-acid sequence of the construct produced	MAHHHHHHAEPLKIALVETLSGPQASTGLLYRAAVLYQLGKINEAGGFNGEKIQILEYDNQGGPVGAADRVKAAIADGAQIIVQGSSSAVAGQITEDVRKYNLRNKGKEVLYLNLGAEALELTGSKCHFYHFRFSPNAAIHFKTVAQGMKDKGILGERAYSINQNYSWGVDVENTVVANAKEIGYEVVDKTLHEVNKIQDFSPYVAKIQAANVDTVFTGNWSNDLLLLMKAASGAGLKAKFATSFLDQPGNIGNAGAIAEGHIVSTPFNPEANGEASMAFAEDYKKVTGHYPSYVEPAAVFGLQLFGEALKNVKPGEGKINTTDIALAIENASVKTPMGDYSMRSDDHQAKFPMVVQEVSKKARIKADGTEYGFLPFKTFTGDESIDPVQESCSMKRPG

**Table 2:** Crystallization

	Crystal 1	Crystal 2 (phasing)	Crystal 3
Method	Vapor diffusion, sitting drop	Vapor diffusion, sitting drop	Vapor diffusion, sitting drop
Temperature (K)	290	290	287
Protein concentration (mg ml^−1^)	25	25	25
Protein buffer composition	20 m*M* HEPES pH 7.0, 300 m*M* NaCl, 5%(*v*/*v*) glycerol, 1 m*M* TCEP	20 m*M* HEPES pH 7.0, 300 m*M* NaCl, 5%(*v*/*v*) glycerol, 1 m*M* TCEP	20 m*M* HEPES pH 7.0, 300 m*M* NaCl, 5%(*v*/*v*) glycerol, 1 m*M* TCEP
Composition of reservoir solution	100 m*M* CHES–NaOH pH 9.5, 30%(*w*/*v*) PEG 3000	1 *M* LiCl, 100 m*M* sodium acetate, 30%(*w*/*v*) PEG 6000	20%(*w*/*v*) PEG 3350, 200 m*M* potassium nitrate
Volume and ratio of drop	0.4 µl:0.4 µl	0.4 µl:0.4 µl	0.5 µl:0.5 µl
Volume of reservoir (µl)	50	50	50
Cryoprotectant	Al’s oil	30 s soak in cryo/phasing solution 1 [4.5 µl reservoir + 0.5 µl 2.5 *M* NaI, 20%(*v*/*v*) ethylene glycol], 30 s soak in cryo/phasing solution 2 [4 µl reservoir + 1 µl 2.5 *M* NaI, 20%(*v*/*v*) ethylene glycol]	Soak in 10 m*M* threonine, 20%(*w*/*v*) PEG 3350, 200 m*M* potassium nitrate, 20% ethylene glycol

**Table 3:** Data collection and processing Values in parentheses are for the outer shell.

	Crystal 1 (monoclinic)	Crystal 2 (phasing data)	Crystal 3 (orthorhombic)
PDB code	4xfk		7jfn
Diffraction source	APS beamline 21-ID-F	Rigaku FR-E+ SuperBright	APS beamline 21-ID-F
Wavelength (Å)	0.97872	1.54178	0.97872
Temperature (K)	100	100	100
Detector	RayoniX MX-225 CCD	Rigaku Saturn 944+ CCD	RayoniX MX-300 CCD
Crystal-to-detector distance (mm)	115	50	250
Rotation range per image (°)	1.0	0.5	1.0
Total rotation range (°)	180	360	150
Exposure time per image (s)	1	30	1
Space group	*P*2_1_	*P*2_1_2_1_2_1_	*P*2_1_2_1_2_1_
*a*, *b*, *c* (Å)	62.12, 46.39, 62.40	60.68, 67.26, 94.49	46.77, 69.39, 120.75
α, β, γ (°)	90, 101.62, 90	90, 90, 90	90, 90, 90
Resolution range (Å)	50–1.30 (1.33–1.30)	50–2.05 (2.10–2.05)	50–1.70 (1.74–1.70)
Total No. of reflections	314986 (22402)	337201 (16407)	243859 (10451)
No. of unique reflections	83917 (6070)	46709 (3419)	43048 (2653)
Completeness (%)	97.9 (96.1)	99.6 (97.7)	97.7 (82.3)
Multiplicity	3.8 (3.7)	7.2 (4.8)	5.7 (3.9)
〈*I*/σ(*I*)〉	17.96 (2.49)	17.64 (3.59)	22.92 (2.57)
*R*_r.i.m._	0.054 (0.605)	0.095 (0.575)	0.042 (0.487)
Overall *B* factor from Wilson plot (Å^2^)	10.36	n/a	33.79

**Table 4:** Structure solution and refinement Values in parentheses are for the outer shell.

	PDB entry 4xfk (monoclinic)	PDB entry 7jfn (orthorhombic)
Resolution range (Å)	30.42–1.30 (1.33–1.30)	34.82–1.70 (1.74–1.70)
Completeness (%)	97.9 (96.1)	97.7 (82.3)
σ Cutoff	*F* > 1.35σ(*F*)	*F* > 1.35σ(*F*)
No. of reflections, working set	81905 (5704)	41102 (2441)
No. of reflections, test set	2009 (139)	1944 (115)
Final *R*_cryst_	0.137 (0.194)	0.156 (0.258)
Final *R*_free_	0.161 (0.248)	0.194 (0.324)
No. of non-H atoms		
Protein	2921	2918
Ion	10	13
Ligand	0	4
Solvent	540	288
Total	3471	3223
R.m.s. deviations from ideal		
Bond lengths (Å)	0.006	0.008
Angles (°)	1.098	0.909
Average *B* factors (Å^2^)		
Protein	12.5	30.4
Ion	18.0	34.3
Ligand	0	55.2
Water	26.6	41.6
Ramachandran plot		
Most favored (%)	94.3	94.3
Allowed (%)	5.7	5.4
Disallowed (%)	0	0.3

## References

[bb1] Abendroth, J., Gardberg, A. S., Robinson, J. I., Christensen, J. S., Staker, B. L., Myler, P. J., Stewart, L. J. & Edwards, T. E. (2011). *J. Struct. Funct. Genomics*, **12**, 83–95. 10.1007/s10969-011-9101-7 PMC3123459 21359836

[bb2] Adams, P. D., Afonine, P. V., Bunkóczi, G., Chen, V. B., Echols, N., Headd, J. J., Hung, L. W., Jain, S., Kapral, G. J., Grosse Kunstleve, R. W., McCoy, A. J., Moriarty, N. W., Oeffner, R. D., Read, R. J., Richardson, D. C., Richardson, J. S., Terwilliger, T. C. & Zwart, P. H. (2011). *Methods*, **55**, 94–106. 10.1016/j.ymeth.2011.07.005 PMC3193589 21821126

[bb3] Agirre, J., Atanasova, M., Bagdonas, H., Ballard, C. B., Baslé, A., Beilsten-Edmands, J., Borges, R. J., Brown, D. G., Burgos-Mármol, J. J., Berrisford, J. M., Bond, P. S., Caballero, I., Catapano, L., Chojnowski, G., Cook, A. G., Cowtan, K. D., Croll, T. I., Debreczeni, J. É., Devenish, N. E., Dodson, E. J., Drevon, T. R., Emsley, P., Evans, G., Evans, P. R., Fando, M., Foadi, J., Fuentes-Montero, L., Garman, E. F., Gerstel, M., Gildea, R. J., Hatti, K., Hekkelman, M. L., Heuser, P., Hoh, S. W., Hough, M. A., Jenkins, H. T., Jiménez, E., Joosten, R. P., Keegan, R. M., Keep, N., Krissinel, E. B., Kolenko, P., Kovalevskiy, O., Lamzin, V. S., Lawson, D. M., Lebedev, A. A., Leslie, A. G. W., Lohkamp, B., Long, F., Malý, M., McCoy, A. J., McNicholas, S. J., Medina, A., Millán, C., Murray, J. W., Murshudov, G. N., Nicholls, R. A., Noble, M. E. M., Oeffner, R., Pannu, N. S., Parkhurst, J. M., Pearce, N., Pereira, J., Perrakis, A., Powell, H. R., Read, R. J., Rigden, D. J., Rochira, W., Sammito, M., Sánchez Rodríguez, F., Sheldrick, G. M., Shelley, K. L., Simkovic, F., Simpkin, A. J., Skubak, P., Sobolev, E., Steiner, R. A., Stevenson, K., Tews, I., Thomas, J. M. H., Thorn, A., Valls, J. T., Uski, V., Usón, I., Vagin, A., Velankar, S., Vollmar, M., Walden, H., Waterman, D., Wilson, K. S., Winn, M. D., Winter, G., Wojdyr, M. & Yamashita, K. (2023). *Acta Cryst.* D**79**, 449–461.

[bb4] Ariza, J., Bosilkovski, M., Cascio, A., Colmenero, J. D., Corbel, M. J., Falagas, M. E., Memish, Z. A., Roushan, M. R., Rubinstein, E., Sipsas, N. V., Solera, J., Young, E. J., Pappas, G., International Society of Chemotherapy & Institute of Continuing Medical Education of Ioannina (2007). *PLoS Med.* **4**, e317. 10.1371/journal.pmed.0040317 PMC2222927 18162038

[bb5] Aslanidis, C. & de Jong, P. J. (1990). *Nucleic Acids Res.* **18**, 6069–6074. 10.1093/nar/18.20.6069 PMC332407 2235490

[bb6] Beard, D. K., Bristol, S., Cosby, K., Davis, A., Manning, C., Perry, L., Snapp, L., Toy, A., Wheeler, K., Young, J., Staker, B., Arakaki, T. L., Abendroth, J., Subramanian, S., Edwards, T. E., Myler, P. J. & Asojo, O. A. (2022). *Acta Cryst.* F**78**, 143. 10.1107/S2053230X22001704 PMC8900735 35234140

[bb7] Beard, D. K., Subramanian, S., Abendroth, J., Dranow, D. M., Edwards, T. E., Myler, P. J. & Asojo, O. A. (2022). *Acta Cryst.* F**78**, 45–51. 10.1107/S2053230X21013455 PMC8805214 35102892

[bb8] Brooks, L., Subramanian, S., Dranow, D. M., Mayclin, S. J., Myler, P. J. & Asojo, O. A. (2022). *Acta Cryst.* F**78**, 306–312. 10.1107/S2053230X22007555 PMC9350836 35924598

[bb9] Bryan, C. M., Bhandari, J., Napuli, A. J., Leibly, D. J., Choi, R., Kelley, A., Van Voorhis, W. C., Edwards, T. E. & Stewart, L. J. (2011). *Acta Cryst.* F**67**, 1010–1014. 10.1107/S1744309111018367 PMC3169394 21904042

[bb10] Choi, R., Kelley, A., Leibly, D., Nakazawa Hewitt, S., Napuli, A. & Van Voorhis, W. (2011). *Acta Cryst.* F**67**, 998–1005. 10.1107/S1744309111017374 PMC3169392 21904040

[bb11] Collaborative Computational Project, Number 4 (1994). *Acta Cryst.* D**50**, 760–763.

[bb12] Davidson, J., Nicholas, K., Young, J., Conrady, D. G., Mayclin, S., Subramanian, S., Staker, B. L., Myler, P. J. & Asojo, O. A. (2022). *Acta Cryst.* F**78**, 25–30. 10.1107/S2053230X21012632 PMC8725002 34981772

[bb13] Ducrotoy, M., Bertu, W. J., Matope, G., Cadmus, S., Conde-Álvarez, R., Gusi, A. M., Welburn, S., Ocholi, R., Blasco, J. M. & Moriyón, I. (2017). *Acta Trop.* **165**, 179–193. 10.1016/j.actatropica.2015.10.023 26551794

[bb14] Emsley, P. & Cowtan, K. (2004). *Acta Cryst.* D**60**, 2126–2132. 10.1107/S0907444904019158 15572765

[bb15] Emsley, P., Lohkamp, B., Scott, W. G. & Cowtan, K. (2010). *Acta Cryst.* D**66**, 486–501. 10.1107/S0907444910007493 PMC2852313 20383002

[bb16] Figueiredo, P. de, Ficht, T. A., Rice-Ficht, A., Rossetti, C. A. & Adams, L. G. (2015). *Am. J. Pathol.* **185**, 1505–1517. 10.1016/j.ajpath.2015.03.003 PMC4450313 25892682

[bb17] Franc, K. A., Krecek, R. C., Häsler, B. N. & Arenas-Gamboa, A. M. (2018). *BMC Public Health*, **18**, 125. 10.1186/s12889-017-5016-y PMC5765637 29325516

[bb18] Godfroid, J., Al Dahouk, S., Pappas, G., Roth, F., Matope, G., Muma, J., Marcotty, T., Pfeiffer, D. & Skjerve, E. (2013). *Comput. Immunol. Microbiol. Infect. Dis.* **36**, 241–248. 10.1016/j.cimid.2012.09.001 23044181

[bb19] Godfroid, J., Garin-Bastuji, B., Saegerman, C. & Blasco, J. M. (2013). *Rev. Sci. Tech. OIE*, **32**, 27–42. 10.20506/rst.32.1.2180 23837363

[bb20] Godfroid, J., Scholz, H. C., Barbier, T., Nicolas, C., Wattiau, P., Fretin, D., Whatmore, A. M., Cloeckaert, A., Blasco, J. M., Moriyon, I., Saegerman, C., Muma, J. B., Al Dahouk, S., Neubauer, H. & Letesson, J. J. (2011). *Prev. Vet. Med.* **102**, 118–131. 10.1016/j.prevetmed.2011.04.007 21571380

[bb21] Gouet, P., Robert, X. & Courcelle, E. (2003). *Nucleic Acids Res.* **31**, 3320–3323. 10.1093/nar/gkg556 PMC168963 12824317

[bb22] Kabsch, W. (2010). *Acta Cryst.* D**66**, 125–132. 10.1107/S0907444909047337 PMC2815665 20124692

[bb23] Krissinel, E. B., Winn, M. D., Ballard, C. C., Ashton, A. W., Patel, P., Potterton, E. A., McNicholas, S. J., Cowtan, K. D. & Emsley, P. (2004). *Acta Cryst.* D**60**, 2250–2255. 10.1107/S0907444904027167 15572778

[bb24] Lee, R. A., Razaz, M. & Hayward, S. (2003). *Bioinformatics*, **19**, 1290–1291. 10.1093/bioinformatics/btg137 12835274

[bb25] Maddy, J., Staker, B. L., Subramanian, S., Abendroth, J., Edwards, T. E., Myler, P. J., Hybiske, K. & Asojo, O. A. (2022). *Acta Cryst.* F**78**, 135–142. 10.1107/S2053230X22002138 PMC8900733 35234139

[bb26] Magnusson, U., Salopek-Sondi, B., Luck, L. A. & Mowbray, S. L. (2004). *J. Biol. Chem.* **279**, 8747–8752. 10.1074/jbc.M311890200 14672931

[bb27] McCoy, A. J., Grosse-Kunstleve, R. W., Adams, P. D., Winn, M. D., Storoni, L. C. & Read, R. J. (2007). *J. Appl. Cryst.* **40**, 658–674. 10.1107/S0021889807021206 PMC2483472 19461840

[bb28] Megersa, B., Biffa, D., Abunna, F., Regassa, A., Godfroid, J. & Skjerve, E. (2011). *Trop. Anim. Health Prod.* **43**, 651–656. 10.1007/s11250-010-9748-2 21088890

[bb29] Moreno, E. (2014). *Front. Microbiol.* **5**, 213. 10.3389/fmicb.2014.00213 PMC4026726 24860561

[bb30] Peck, D. & Bruce, M. (2017). *Rev. Sci. Tech. OIE*, **36**, 291–302. 10.20506/rst.36.1.2629 28926008

[bb31] Porter, I., Neal, T., Walker, Z., Hayes, D., Fowler, K., Billups, N., Rhoades, A., Smith, C., Smith, K., Staker, B. L., Dranow, D. M., Mayclin, S. J., Subramanian, S., Edwards, T. E., Myler, P. J. & Asojo, O. A. (2022). *Acta Cryst.* F**78**, 31–38. 10.1107/S2053230X21013078 PMC8725004 34981773

[bb32] Qi, G., Lee, R. & Hayward, S. (2005). *Bioinformatics*, **21**, 2832–2838. 10.1093/bioinformatics/bti420 15802286

[bb33] Riedel, S. (2004). *Bayl. Univ. Med. Cent. Proc.* **17**, 400–406. 10.1080/08998280.2004.11928002 PMC1200679 16200127

[bb34] Robert, X. & Gouet, P. (2014). *Nucleic Acids Res.* **42**, W320–W324. 10.1093/nar/gku316 PMC4086106 24753421

[bb35] Rossetti, C. A., Maurizio, E. & Rossi, U. A. (2022). *Front. Vet. Sci.* **9**, 887671. 10.3389/fvets.2022.887671 PMC9133814 35647101

[bb36] Sack, J. S., Trakhanov, S. D., Tsigannik, I. H. & Quiocho, F. A. (1989). *J. Mol. Biol.* **206**, 193–207. 10.1016/0022-2836(89)90532-9 2649683

[bb37] Serbzhinskiy, D. A., Clifton, M. C., Sankaran, B., Staker, B. L., Edwards, T. E. & Myler, P. J. (2015). *Acta Cryst.* F**71**, 594–599. 10.1107/S2053230X15004677 PMC4427170 25945714

[bb38] Studier, F. W. (2005). *Protein Expr. Purif.* **41**, 207–234. 10.1016/j.pep.2005.01.016 15915565

[bb41] Trakhanov, S., Vyas, N. K., Luecke, H., Kristensen, D. M., Ma, J. & Quiocho, F. A. (2005). *Biochemistry*, **44**, 6597–6608. 10.1021/bi047302o 15850393

[bb39] Williams, C. J., Headd, J. J., Moriarty, N. W., Prisant, M. G., Videau, L. L., Deis, L. N., Verma, V., Keedy, D. A., Hintze, B. J., Chen, V. B., Jain, S., Lewis, S. M., Arendall, W. B., Snoeyink, J., Adams, P. D., Lovell, S. C., Richardson, J. S. & Richardson, J. S. (2018). *Protein Sci.* **27**, 293–315. 10.1002/pro.3330 PMC5734394 29067766

[bb40] Winn, M. D., Ballard, C. C., Cowtan, K. D., Dodson, E. J., Emsley, P., Evans, P. R., Keegan, R. M., Krissinel, E. B., Leslie, A. G. W., McCoy, A., McNicholas, S. J., Murshudov, G. N., Pannu, N. S., Potterton, E. A., Powell, H. R., Read, R. J., Vagin, A. & Wilson, K. S. (2011). *Acta Cryst.* D**67**, 235–242. 10.1107/S0907444910045749 PMC3069738 21460441

