# Supplementary material for: Structures of Brucella ovis leucine-, isoleucine-, valine-, threonine- and alanine-binding protein reveal a conformationally flexible peptide-binding cavity
Source: Acta Crystallogr F Struct Biol Commun. 2024 Aug 23;80(Pt 9):193–9. doi: 10.1107/S2053230X24007027 (PMC11376275; doi:10.1107/S2053230X24007027)
Supplement: Supplementary file 1 [file f-80-00193-sup1.pdf]

STRUCTURAL BIOLOGY
COMMUNICATIONS

Volume 80 (2024)

Supporting information for article:

Structures of *Brucella ovis* leucine-, isoleucine-, valine-, threonine- and alanine-binding protein reveal a conformationally flexible peptide-binding cavity

Graham Chakafana, Reghan Boswell, Andrew Chandler, Krishelle A. Jackson, Senai Neblett, Tyler Postal, Sandhya Subramanian, Jan Abendroth, Peter J. Myler and Oluwatoyin A. Asojo

Ligands bound in the binding site are crystallization artifacts and usurped from the crystallization solution.

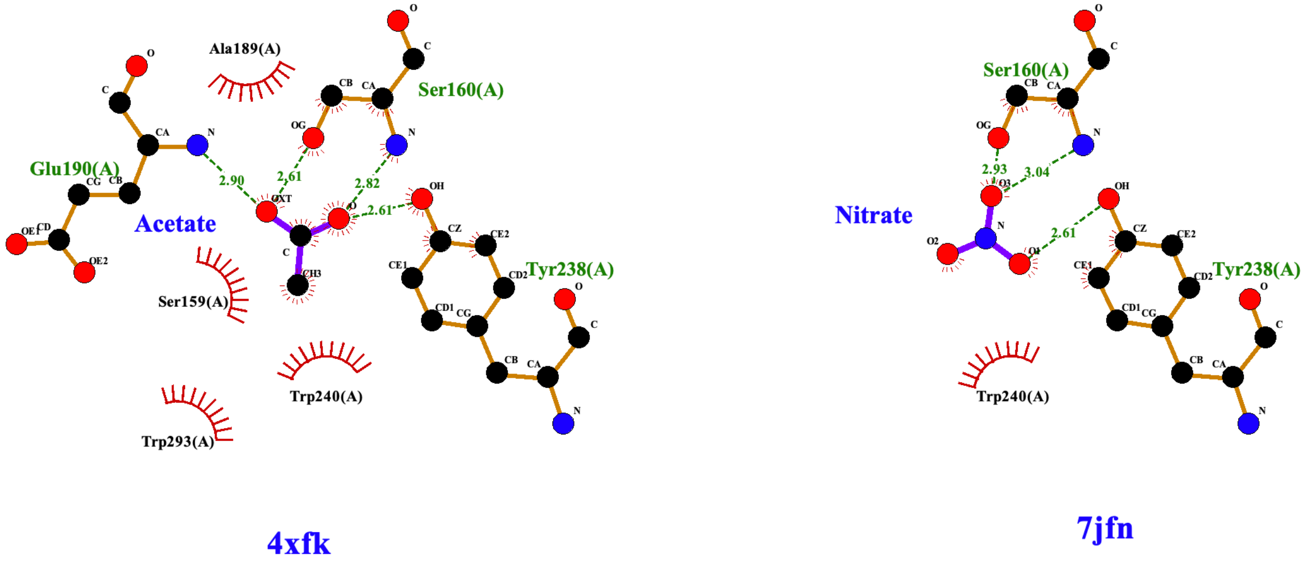

Figure S1a: Ligplus map of the interactions of acetate and nitrate atoms in the binding cavities of 4xfk and 7jfn, respectively.

These ligands have well-ordered electron density maps, as shown below.

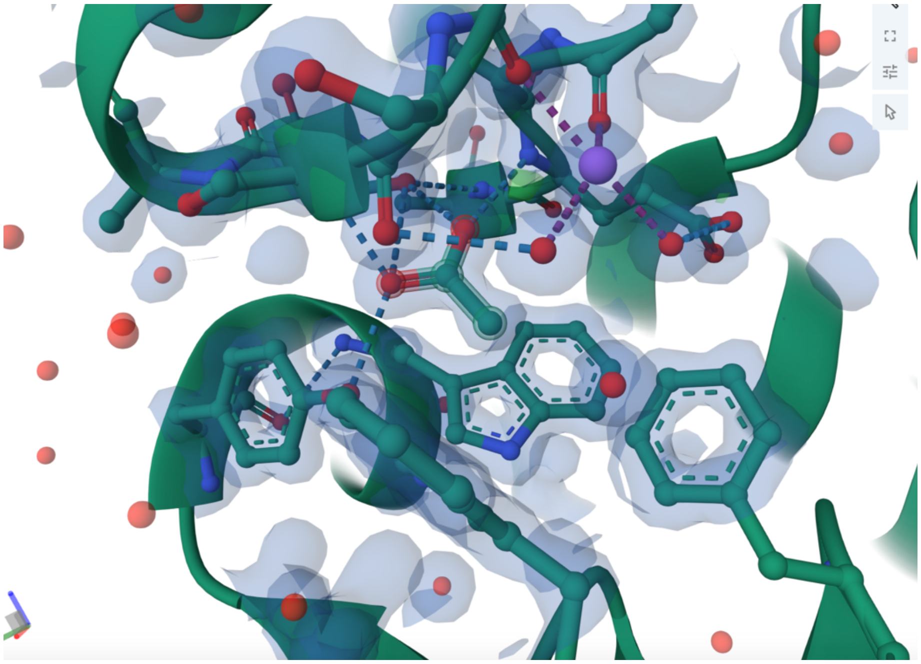

Figure S1b Acetate in the binding cavity of PDB entry 4xfk. Image generated with pdb viewer and 2Fo-F map electron density contoured at 1.6σ

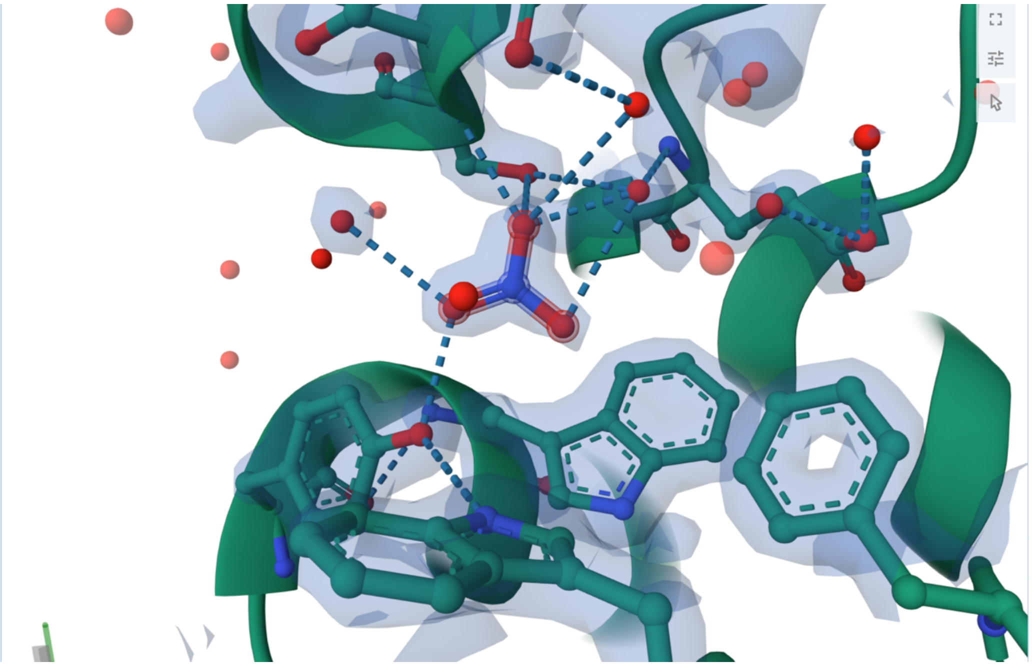

Figure S1c: Nitrate in the binding cavity of PDB entry 7jfn. Image generated with pdb viewer and 2Fo-F map electron density contoured at 1.74σ

S2. DynDom Analysis

Domain Pairs

Property	Value
Fixed Domain (blue)	1
Moving Domain (red)	2
Rotation Angle (deg)	12.0
Translation (A)	0.2
Closure (%)	99.1
Bending Residues (green)	211 - 212<br>316 - 322<br>326 - 335<br>339 - 340<br>426 - 429<br>448 - 449<br>456 - 457
Bending Region Analysis	

Dynamic Contact Graph

Conformer 1 Contact:
Residue 1 ---› Residue 2

Conformer 2 Contact:
Residue 2 ---› Residue 1

Movement classification: Hinge

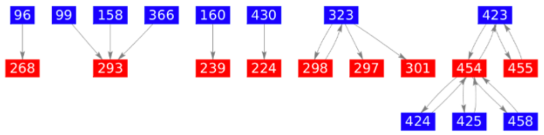

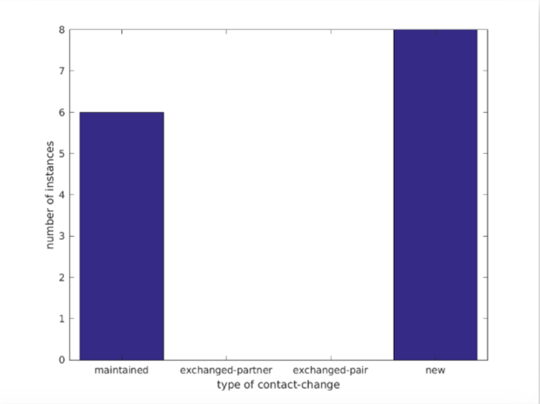

```
           ______________________________________________________________________________________
4XFK(A) :  AEPLKIALVETLSGPQASTGLLYRAAVLYQLGKINEAGGFNGEKIQILEYDNQGGPVGAADRVKAAIADGAQIIVQGSS
        :
7JFN(A) :  AEPLKIALVETLSGPQASTGLLYRAAVLYQLGKINEAGGFNGEKIQILEYDNQGGPVGAADRVKAAIADGAQIIVQGSS
           ______________________________________________________________________________________

           ________211___________________________________________________________________________
4XFK(A) :  FYHFRFSPNAAIRFKTVAQGMKDKGILGERAYSINQNYSWGVDVENTVVANAKEIGYEVVDKTLHEVNKIQDFSPYVAK
        :
7JFN(A) :  FYHFRFSPNAAIRFKTVAQGMKDKGILGERAYSINQNYSWGVDVENTVVANAKEIGYEVVDKTLHEVNKIQDFSPYVAK
           ________211___________________________________________________________________________

           ___326__________339___________________________________________________________________
4XFK(A) :  PGNIGNAGAIAEGHIVSTPFNPEANGEASMAFAEDYKKVTGHYPSYVEPAAVFGLQLFGEALKNVKPGEGKINTTDIAL
        :
7JFN(A) :  PGNIGNAGAIAEGHIVSTPFNPEANGEASMAFAEDYKKVTGHYPSYVEPAAVFGLQLFGEALKNVKPGEGKINTTDIAL
           ___326__________339___________________________________________________________________

           _____448_____456_______________
4XFK(A) :  GTEYGFLPFKTFTGDESIDPVQESCSMKRPG
        :
7JFN(A) :  GTEYGFLPFKTFTGDESIDPVQESCSMKRP*
           _____448_____456_______________
```

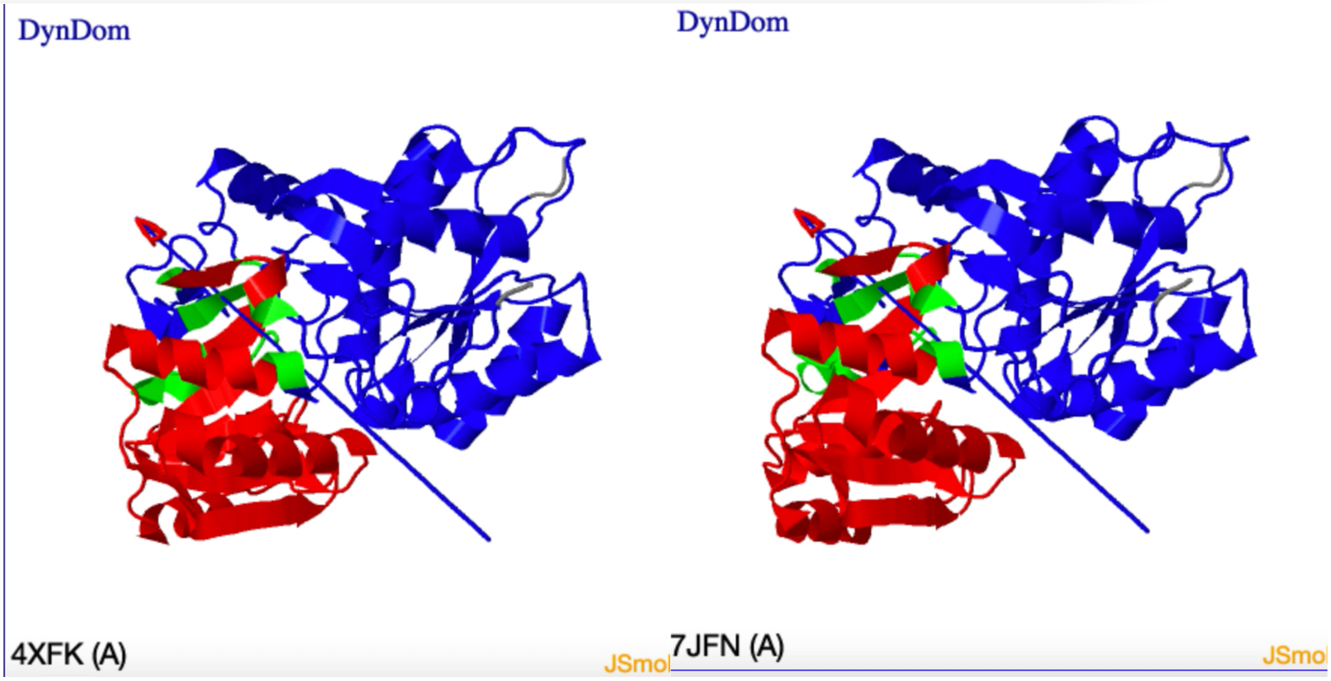

The conformational plasticity is more evident in the movie than in still images.
